# Supplementary figures and images for: Evaluating the efficacy and safety of mavacamten in hypertrophic cardiomyopathy: A systematic review and meta-analysis focusing on qualitative assessment, biomarkers, and cardiac imaging
Source: PLoS One. 2024 Apr 18;19(4):e0301704. doi: 10.1371/journal.pone.0301704 (PMC11025865; doi:10.1371/journal.pone.0301704)

S2 Fig: Risk of Bias of the included studies


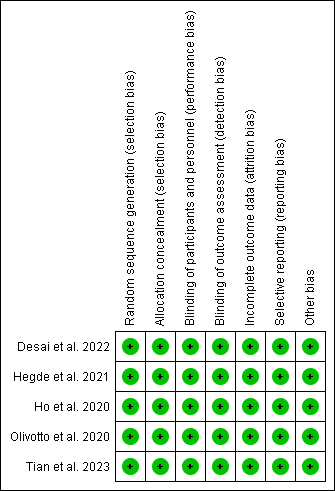

Supplement: S2 Fig — (DOCX) [file pone.0301704.s002.docx]
