# Supplementary material for: Evaluating the efficacy and safety of mavacamten in hypertrophic cardiomyopathy: A systematic review and meta-analysis focusing on qualitative assessment, biomarkers, and cardiac imaging
Source: PLoS One. 2024 Apr 18;19(4):e0301704. doi: 10.1371/journal.pone.0301704 (PMC11025865; doi:10.1371/journal.pone.0301704)
Supplement: S1 Appendix — (DOCX) [file pone.0301704.s003.docx]

|  | **Mavacamten - Event** | **Mavacamten - Total** | **Placebo - Event** | **Placebo - Total** | **Odds Ratio, 95% CI** |
| --- | --- | --- | --- | --- | --- |
| **NYHA** |  |  |  |  |  |
| Olivotto et al. | 80 | 123 | 40 | 128 | 4.09 (2.42, 6.93) |
| Desai et al. | 35 | 56 | 12 | 56 | 6.11 (2.65, 14.11) |
| Tian et al. | 32 | 54 | 4 | 27 | 8.36 (2.54, 27.56) |

|  | **Mean Difference** | **SE** | **Mean Difference, 95% CI** |
| --- | --- | --- | --- |
| **KCCQ-CSS** |  |  |  |
| Ho et al. | 0.97 | 3.319 | 0.97 (-5.54, 7.48) |
| Olivotto et al. | 9.1 | 1.84 | 9.1 (5.49, 12.71) |
| Desai et al. | 9.4 | 2.32 | 9.4 (4.85, 13.95) |
| Tian et al. | 10.2 | 2.98 | 10.2 (4.36, 16.04) |
| **LAVI** |  |  |  |
| Desai et al. | -4.4 | 1.377 | -4.4 (-7.10 -, -1.70) |
| Hegde et al. | -7.5 | 1 | -7.5 (-9.46 -, -5.54) |
| Ho et al. | -2.21 | 2.587 | -2.21 (-7.28, 2.86) |
| **LVOT at Rest** |  |  |  |
| Desai et al. | -33.4 | 17.04 | -33.4 (-66.80, -0.00) |
| Tian et al. | -55 | 7.19 | -55 (-69.05, -40.91) |
| **LVOT Post Exercise** |  |  |  |
| Desai et al. | -47.6 | 5.41 | -47.6 (-58.20, -37.00) |
| Tian et al. | -70.3 | 9.87 | -70.3 (-89.64, -50.96) |
| **LVOT Post Valsalva** |  |  |  |
| Olivotto et al. | -35.6 | 3.85 | -35.6 (-43.15, -28.05) |
| Desai et al. | -37.2 | 5.59 | -37.2 (-48.16, -26.24) |
| **LVEF** |  |  |  |
| Desai et al. | -4 | 0.765 | -4 (-5.50, -2.50) |
| Olivotto et al. | -4 | 0.765 | -4 (-5.50, -2.50) |
| Ho et al. | 1.78 | 2.158 | 1.78 (-2.45, 6.01) |
| **NT-Pro BNP** |  |  |  |
| Olivotto et al. | 0.202 | 0.018 | 0.20 (0.17, 0.24) |
| Tian et al. | 0.18 | 0.028 | 0.18 (0.13, 0.23) |
| **Cardiac Troponin- I** |  |  |  |
| Desai et al. | 0.53 | 0.074 | 0.53 (0.38,0.68) |
| Olivotto et al. | 0.589 | 0.05 | 0.59 (0.49, 0.69) |
