# Supplementary material for: Evaluating the efficacy and safety of mavacamten in hypertrophic cardiomyopathy: A systematic review and meta-analysis focusing on qualitative assessment, biomarkers, and cardiac imaging
Source: PLoS One. 2024 Apr 18;19(4):e0301704. doi: 10.1371/journal.pone.0301704 (PMC11025865; doi:10.1371/journal.pone.0301704)
Supplement: Attachment — Submitted filename: Response to reviewer comments.docx [file pone.0301704.s004.docx]

Response to Reviewers comments

| **Reviewer 1** | **Author’s Response** |
| --- | --- |
| A nicely presented review with relevant RCTs and trials for mavacamtem in the setting of HOCM. I'would suggest adding speckle tracking imaging data, where and if available (GLS%), to echocardiographic parameters. Furthermore, I would add a section specific fo CMR data. | Thank you for this feedback. We totally agree with you on including any relevant data on speckle tracking imaging data to understand more on the myocardial wall strain. However, since the included RCTs measured the parameters as a form of Doppler ECHO we primarily included that as we were not able to find any data available on speckle tracking or CMR. |
| **Reviewer 2** | |
| The claim of including five phase 3 RCTs on mavacamten and excluding all subanalyses of RCTs is contradicted by the inclusion of the study by Hedge et al., which is a subanalysis of EXPLORER-HCM (Olivotto et al.). While the inclusion of the Hedge et al. subanalysis is considered reasonable due to its unique information on echocardiographic data, the claim of encompassing five RCTs should be thoroughly reviewed throughout the text. | Thank you for your comment. We have added one sentence under inclusion criteria justifying the need to include any sub analysis if they provided with extra information from the parent RCTs. |
| In the discussion, it is recommended to cite the other two recently published meta-analyses on mavacamten which include the same four trials as the present study (DOI: 10.1007/s10741-023-10375-6; DOI: 10.1186/s43044-023-00427-5). Emphasis should be placed on elucidating the novelty of the current meta-analysis compared to these prior studies. | We have added the novelty of our article under the conclusion section and comparing it with the two studies mentioned by the reviewer. |
| Replace "pro-BNP" with "NT-proBNP." | We have made changes accordingly. |
| Ensure that acronyms are spelled out in their extended form the first time they are cited in the text (e.g., "NT-proBNP", "hs-cTnI", and so forth). | We have made changes accordingly. |
